# Lichen Planus Pigmentosus: A Clinicopathological Study From Northeast India

**DOI:** 10.7759/cureus.74627

**Published:** 2024-11-27

**Authors:** Adahra P Beso, Mary Z Chhangte, Biswajit Dey, Shikha Verma

**Affiliations:** 1 Pathology, State Cancer Institute, Gauhati Medical College and Hospital (GMCH), Guwahati, IND; 2 Dermatology, All India Institute of Medical Sciences, Guwahati, Guwahati, IND; 3 Pathology and Laboratory Medicine, North Eastern Indira Gandhi Regional Institute of Health and Medical Sciences (NEIGRIHMS), Shillong, IND; 4 Dermatology, North Eastern Indira Gandhi Regional Institute of Health and Medical Sciences (NEIGRIHMS), Shillong, IND

**Keywords:** diffuse, histopathology (hp), lichen planus pigmentosus, melanin incontinence, skin pigmentation

## Abstract

Introduction: Lichen planus pigmentosus (LPP) is an uncommon variant of lichen planus, characterized by the insidious onset of dark brown to gray pigmented macules, mainly in sun-exposed areas and flexural folds. It is mainly reported in Indian, Latino, American, and Middle Eastern patients. This paper aims to document the clinicopathological characteristics of LPP.

Materials and methods: A five-year retrospective study from January 2017 to December 2021 analyzed 42 patients diagnosed with LPP who presented with idiopathic, itchy/asymptomatic, hyperpigmented/violaceous macules at the Dermatology outpatient department. The study excluded those with melasma, post-inflammatory pigmentation, or cases where a skin biopsy was unavailable. Demographic, clinical, and histopathological data, including age, sex, residence, site of involvement, pigmentation pattern, and biopsy results, were reviewed. Routine hematoxylin-eosin staining was performed on all biopsies, with special stains such as Masson's trichrome and Congo red used in selected cases to assess fibrosis and to rule out amyloid.

Results: The study involved 42 patients, with a higher prevalence in female patients, 29 (69.0%) compared to 13 (30.9%) male patients, and an average age of 34.2 years. The majority of patients were from urban areas (28, 66.7%), with the most common sites of involvement being the head and neck (14, 33.3%), upper limbs (nine, 21.4%), and back (eight, 19%). Pigmentation patterns were predominantly diffuse (29, 69%), with less common patterns including reticular (seven, 16.7%) and blotchy (four, 9.5%). Histopathological findings included orthokeratosis, epidermal thinning, and melanin incontinence, with Masson's trichrome staining indicating fibrosis in nine (21.4%) cases. Civatte bodies were present in 33 (78.3%) cases. Direct immunofluorescence showed IgM positivity in one of six cases.

Conclusion: LPP is a common pigmentary disorder characterized by persistent, asymptomatic, slaty-gray pigmentation, mainly in sun-exposed areas. Histopathologically, it features orthokeratosis, hypergranulosis, dense dermal lymphocytic infiltrate, melanin incontinence, and frequent Civatte bodies.

## Introduction

Lichen planus pigmentosus (LPP) is an uncommon variant of lichen planus, characterized by the insidious onset of dark-brown to gray pigmented macules in sun-exposed areas and flexural folds with or without pruritus. It is a chronic, relapsing condition characterized by worsening symptoms alternating with periods of remission, during which symptoms may decrease or disappear [1]. Its evolution is chronic and progressive, marked by unpredictable remissions and exacerbations [2].

The term "lichen planus pigmentosus" was first introduced by Shima in 1956, initially thought to be a variant of lichen planus [3]. Bhutani et al. later reported it in India and described the clinical and histopathological features in 40 patients in 1974 [4]. In 2003, Kanwar et al. conducted a significant study in India with 124 patients, detailing the clinical, epidemiological, and histopathological aspects of LPP [5].

LPP typically presents as pigmentation of insidious onset without inflammation or preceding raised lesions. It is usually asymptomatic but may occasionally cause mild pruritus [6]. The histopathological features of LPP are well documented and include epidermal atrophy, basal cell degeneration, band-like infiltrates, and melanophages in the upper dermis [7].

Most reports of LPP come from Japan, Korea, the Middle East, and Latin America, with a notable absence of studies from North-East India despite the significant research conducted in other regions of India [4,5,7,8]. This study aims to investigate the clinical, epidemiological, and histopathological features of LPP at a tertiary care center in North-East India, which serves patients across the region.

## Materials and methods

A retrospective study was conducted over five years, from January 2017 to December 2021, at the North Eastern Indira Gandhi Regional Institute of Health and Medical Sciences. The inclusion criteria included all the patients who presented at the Dermatology OPD with idiopathic, itchy/asymptomatic, hyperpigmented, or violaceous macules with subsequent skin biopsy diagnosed as LPP. Patients presenting with melasma or post-inflammatory pigmentation, as well as those clinically suspected cases of LPP where skin biopsy correlation was not available, were excluded from the study. A total of 42 cases were included in the study.

The records of these patients were analyzed for demographic, clinical, and histopathological features. Demographic data included age, sex, and place of residence (urban versus rural). The predominant sites of involvement and pigmentation patterns were recorded from photographs taken at the presentation in the OPD. All the patients where histopathological features of LPP were seen were included in the study, and clinical and histopathological findings were noted. Routine hematoxylin-eosin stain was done in all cases.

Special stains, such as Masson's trichrome stain to assess fibrosis and Congo red to rule out amyloid, were performed in selected cases. Any other associated systemic or dermatological diseases were also noted.

## Results

A total of 42 patients were included in the study. Female patients (29, 69.0%) outnumbered male patients (13, 30.9%) with a ratio of 2:1. The mean age of patients was 34.2 years. Nineteen patients were from the age group of 10-29 years (45.2%), 17 patients were from the 30-49 years age group (40.5%), and the remaining six patients were from the 50-69 years age group (14.3%). The majority of our patients (28, 66.7%) were from urban areas, and the rest (14, 33.3%) were from rural areas. Assessment of the predominant site of involvement revealed that 14 (33.3%) patients exhibited head and neck involvement, followed by the upper limbs in nine (21.4%) patients and the back in eight (19%) patients. The other sites involved were the chest in seven (16.7%) patients, abdomen in two (4.8%), flexures in one (2.4%), and lower limbs in one (2.4%) patient (Table 1).

**Table 1:** Clinical features of the cases of LPP LPP: Lichen planus pigmentosus

Clinical Features	No. of Patients (n=42)	Percentage (%)
Age group		
10-29	19	45.2
30-49	17	40.5
50-69	06	14.3
Site of presentation		
Head & neck	14	33.3
Upper limbs	09	21.4
Back	08	19.0
Chest	07	16.7
Abdomen	02	4.8
Flexures (axilla/inframammary areas)	01	2.4
Lower limbs	01	2.4
Pattern of pigmentation		
Diffuse	29	69.0
Reticular	07	16.7
Blotchy	04	9.5
Perifollicular	01	2.4
Localized	01	2.4
Residence		
Urban	28	66.7
Rural	14	33.3

**Figure 1:**
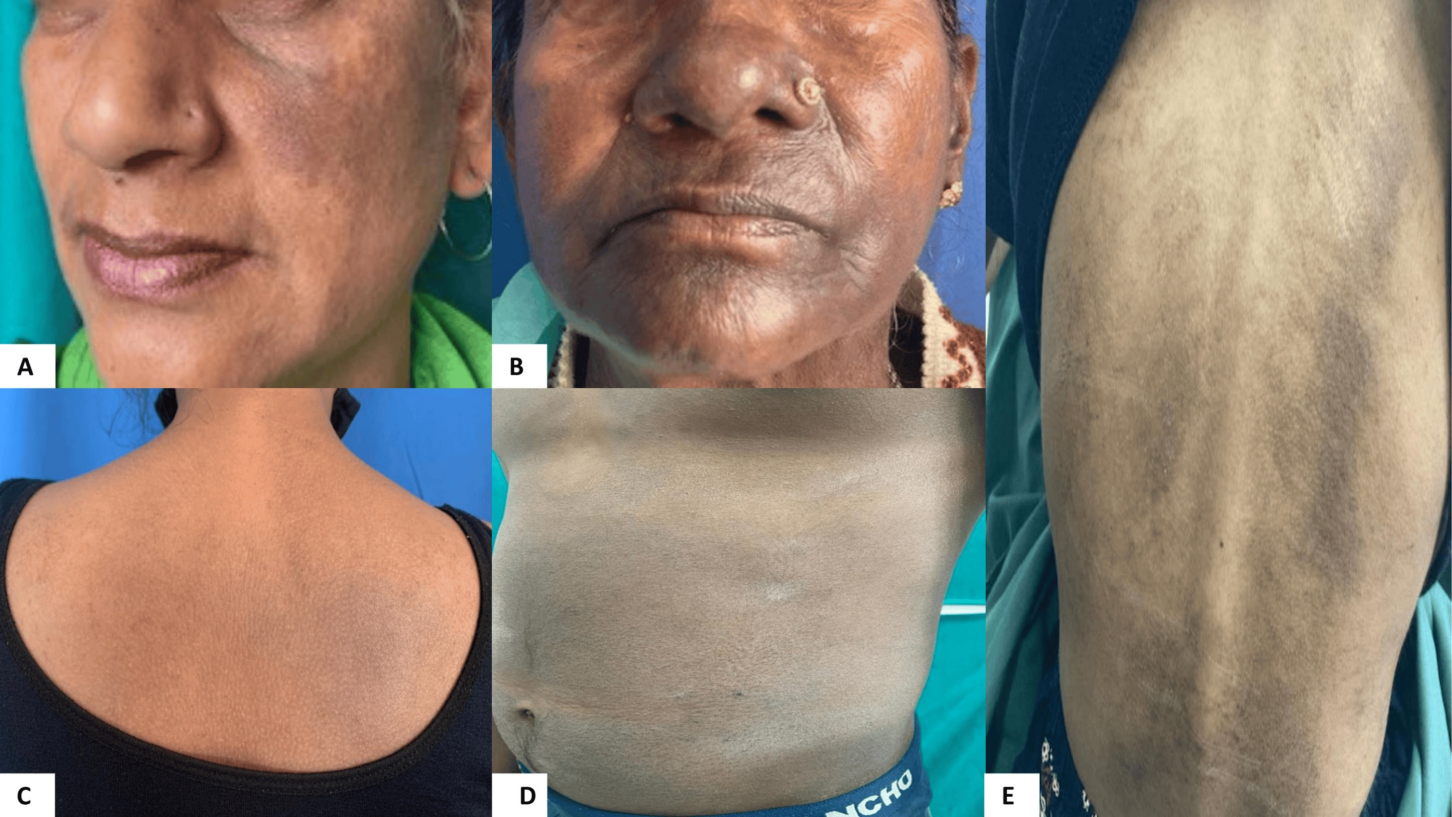
Different patterns of pigmentation of LPP. (A) Localized pattern of LPP on one side of the face. (B) Blotchy pattern of LPP. (C) Perifollicular pattern of LPP. (D) Diffuse pattern of LPP. (E) Burnt-out stage of LPP LPP: Lichen planus pigmentosus

The patterns of pigmentation seen were diffuse in the majority of the cases, accounting for 29 (69%), reticular in seven (16.7%), blotchy in four (9.5%), perifollicular in one (2.4%), and localized in one (2.4%) of the patients (Table 1) (Figure 1). Our study did not see other atypical pigmentation patterns like annular or gyrate.

A review of the histopathological findings showed orthokeratosis, epidermal thinning, hypergranulosis, obliteration of dermo-epidermal junction, pigmentary incontinence with melanophages, basal cell vacuolization, Civatte bodies, and inflammatory infiltrate (Table 2).

**Table 2:** Pathological features of LPP LPP: Lichen planus pigmentosus

Findings	Histopathological Features (n=42)	Percentage (%)
Epidermis		
Orthokeratosis	36	85.7
Epidermal thinning	17	40.5
Hypergranulosis	31	73.8
Civatte bodies	33	78.6
Dermo-epidermal junction		
Dermo-epidermal junction obliteration	6	14.3
Basal cell vacuolization	11	26.2
Dermis		
Pigmentary incontinence (melanophages)	36	85.7
Mild inflammatory infiltrate	18	42.9
Moderate to marked inflammatory infiltrate	24	57.1
Perivascular pattern of inflammatory infiltrate	22	52.4
Periadnexal pattern of inflammatory infiltrate	20	47.6
Fibrosis	09	21.4
Direct immunofluorescence (n=6)		
Positive	01	16.7
Negative	05	83.3

**Figure 2:**
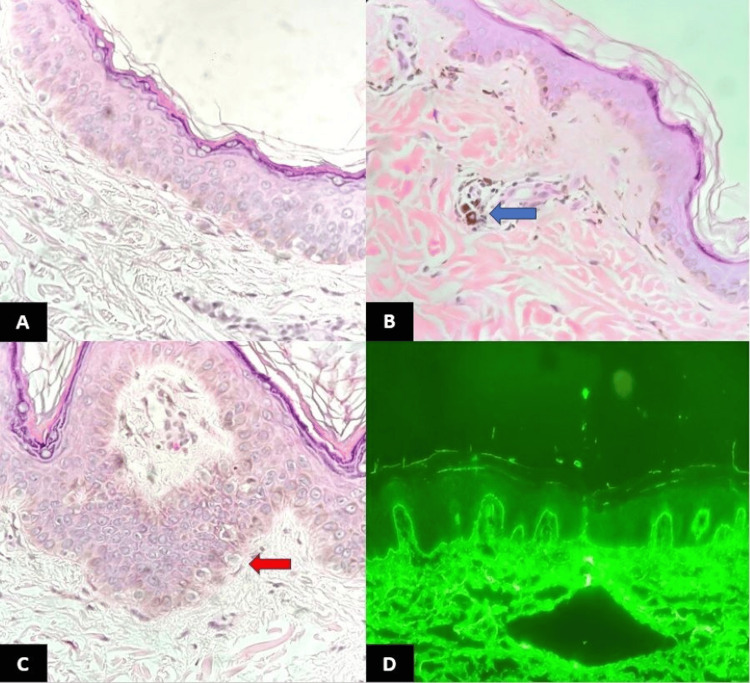
Histopathology and immunofluorescence images of LPP. (A) Skin biopsy in LPP showing thinning of the epidermis with loss of rete ridges (H&E, 40×). (B) Skin biopsy of LPP showing a dermal lymphocytic infiltrate with melanin incontinence (blue arrow) (H&E, 40×). (C) Basal cell vacuolization in LPP (red arrow) (H&E, 40×). (D) Direct immunofluorescence showing IgM in the dermo-epidermal junction (IF, 20×) LPP: Lichen planus pigmentosus; IF: immunofluorescence; H&E: hematoxylin and eosin

Masson's trichrome highlighted fibrosis in nine (21.4%) patients, implying that the lesions were in the receding phase of the disease in these patients. Direct immunofluorescence was performed on six cases, and only one showed IgM positivity at the dermo-epidermal junction in a shaggy pattern (Figure 2).

Three of the 42 patients had associated clinical features including Lichen planopilaris, frontal fibrosing alopecia and oral lichen planus.

## Discussion

LPP is a common pigmentary condition with distinct clinical and histological features, as observed in the present study. It is characterized by persistent, asymptomatic, slaty-gray pigmentation primarily affecting photo-exposed areas, particularly the face and neck, and occasionally extending to the upper limbs [1]. Over time, involvement may also extend to the upper extremities and the upper parts of the back and trunk [9]. Lesions are typically bilaterally symmetrical and predominantly located in sun-exposed areas. These observations are consistent with findings reported by Bhutani et al. [4] and Vega et al. [10], which align with the results of our study.

Classically, the pigmentation pattern is "actinic" with symmetric and diffuse pigmentation, commonly seen in dark‑skinned individuals and can also rarely present as a macular pigmentation of the flexural region in the lighter‑skinned population [1]. The pigmentation is dermal, and there are no signs of inflammation on clinical presentation [6]. Although the pigmentation is usually diffuse, blotchy, reticular, perifollicular, inversus, segmental, annular, and gyrate patterns have also been described [5,11]. There are isolated case reports of linear unilateral hyperpigmentation in the extremities (Blaschkoid) and segmental patterns on the trunk [6]. Kanwar et al. revealed diffuse (77.4%), followed by reticular (9.7%), blotchy (7.3%), and perifollicular (5.6%) patterns of pigmentation [5]. The pigmentation patterns observed in the present study were predominantly diffuse, followed by reticular, blotchy, perifollicular, and localized presenting on one side of the face. Other patterns of pigmentation were not encountered in our study.

Consistent with other studies, our research found that in 40% of cases, symptoms first began in the third or fourth decade of life [5]. Vega et al. reported a female preponderance similar to that observed in the present study [10]. On the contrary, Bhutani et al. observed no difference in sex distribution in their patients [4].

LPP is insidious in onset and has a chronic relapsing course. Some patients continue to develop new lesions while the old ones gradually enlarge with a deepening in color. Generally, the lesions are asymptomatic, although mild pruritus and burning sensations are present in about one-third of patients [9]. Consistent with other studies, our research found no preceding or associated erythema around the lesions [4,5].

The majority of our patients were from urban areas (66.7%) compared to rural areas (33.3%). This finding corroborates with the increased prevalence of lichen planus and its variants among the urban population. Factors contributing to this causation require further studies [12].

The histopathology of LPP forms a continuous spectrum, with the earliest lesions showing marked inflammation at the interface, which later subsides, leaving behind the characteristic dermal pigmentation in older lesions [6], which was also noted in our study.

The inflammatory phase is characterized by a dense band-like or perivascular lymphocytic inflammatory infiltrate in the papillary dermis with prominent basal-vacuolar degeneration. In older lesions or the burnt-out inflammatory phase, marked melanin incontinence with many interstitial and perivascular melanophages is seen with only a mild superficial perivascular lymphocytic infiltrate and focal to absent basal‑vacuolar degeneration [6]. The epidermis is usually atrophic, with orthohyperkeratosis in some cases [1]. The histological findings correlated with the clinical picture, i.e., the majority of the cases presented with hyperpigmentation rather than the typical violaceous skin lesion.

Civatte bodies are a common finding in LPP [1,4]. In the present study, epidermal thinning was seen in less than 50% of cases; however, most cases showed orthokeratosis and hypergranulosis. Basal vacuolization was observed in a small number of cases, while melanin incontinence was a prominent finding in the majority of cases. A moderate to marked inflammatory infiltrate was observed, with the majority located in the perivascular regions. Similar to other studies, Civatte bodies were observed in most cases in the present study.

The comparative analysis of different demographic, clinical, and histopathological findings of the present study with those of other similar studies on LPP is summarized in Table 3.

**Table 3:** Comparison of clinical and histopathological findings with other similar studies on LPP LPP: Lichen planus pigmentosus

Study	Age Range	Gender Preponderance	Common Sites of Involvement	Most Common Pigmentation Pattern	Histopathology
Bhutani et al. (1974) [4]	12-50 years	-	Face and upper extremities	Diffuse	Basal cell degeneration, pigmentary incontinence, and colloid bodies
Kanwar et al. (2003) [5]	13-62 years	Female	Face and neck	Diffuse	Epidermal changes, basal cell degeneration, and pigmentary incontinence
Mendiratta et al. (2019) [11]	18-54 years	Female	Face and neck	Diffuse	-
Present study (2024)	10-69 years	Female	Head and neck	Diffuse	Epidermal changes, pigmentary incontinence, and Civatte bodies

Immune deposits are not commonly found in LPP. Approximately 15% of cases show immune deposits. The patterns seen include IgM and, less commonly, C3 deposits along the dermo-epidermal junction [6]. In the present study, only one case showed a deposit of IgM at the dermo-epidermal junction.

Limitations

The limitations of the study include its retrospective nature and small sample size. As a retrospective study, it was not possible to obtain detailed information regarding the prior use of topical agents. Additionally, the small sample size was due to the study period coinciding with the COVID-19 pandemic.

## Conclusions

LPP is a prevalent pigmentary disorder with distinctive clinical and histological characteristics. This study highlights the clinical and histopathological patterns of LPP observed at a tertiary care hospital in Northeast India. The findings are similar to the studies in other parts of the country. LPP typically presents as persistent, asymptomatic, slaty-gray pigmentation predominantly on sun-exposed areas such as the face and neck, with lesions often bilaterally symmetrical. Pigmentation patterns vary but commonly include diffuse, reticular, blotchy, and perifollicular types. Histopathologically, it features orthokeratosis, hypergranulosis, dense dermal lymphocytic infiltrate, melanin incontinence, and frequent Civatte bodies.

The present study shows a higher prevalence in the urban population, which warrants further studies to determine its relation to the causative factors in LPP. Further prospective studies with larger sample sizes are required to better understand the disease and, hence, better treatment options.
